# Ostéogenèse imparfaite: à propos de quatre cas à Ouagadougou (Burkina Faso)

**DOI:** 10.11604/pamj.2015.22.69.6299

**Published:** 2015-09-28

**Authors:** Aïssata Kaboré, Aissata Cissé, Caroline Yonaba, Hamidou Savadogo, Sylvie Armelle Ouédraogo, Lassina Dao, Sonia Kaboret, Kisito Nagalo, Fla Koueta, Emile Bandré, Diarra Yé, Ludovic Kam

**Affiliations:** 1 Service de Pédiatrie Médicale, Centre Hospitalier Universitaire Pédiatrique Charles de Gaulle de Ouagadougou, Burkina Faso; 2 Service de Pédiatrie, Centre Hospitalier Universitaire Yalgado Ouedraogo, Ouagadougou, Burkina Faso; 3 Service de Chirurgie, Centre Hospitalier Universitaire Pédiatrique Charles de Gaulle de Ouagadougou, Burkina Faso

**Keywords:** Ostéogenèse imparfaite, anomalies osseuses, Burkina-Faso, Osteogenesis imperfecta, bone abnormalities, Burkina-Faso

## Abstract

L'ostéogenèse imparfaite (OI) regroupe un ensemble d'affections constitutionnelles de gravité variable dû à une anomalie de la production du collagène et de la matrice de l'os entraînant une fragilité osseuse. La présente étude rapporte quatre cas d'ostéogenèse imparfaite suivis aux Centres Hospitaliers Universitaires Charles de Gaulle et Yalgado Ouédraogo. Le but de ce travail était d'analyser les aspects cliniques, thérapeutiques et évolutifs de la maladie. Cette étude souligne la nécessité d'améliorer la prise en charge de cette maladie rare mais non exceptionnelle et handicapante.

## Introduction

L'ostéogenèse imparfaite (OI) est l'une des affections génétiques les plus fréquentes du tissu conjonctif qui touche principalement les os et entraînant des fractures à répétition [1, 2]. Sa fréquence est estimée entre 1/10 000 et 1/20 000 naissances vivantes [2, 3]. C'est une maladie qui est liée à une anomalie des gènes codant pour le collagène de type 1. L'ostéogenèse imparfaite est classée en sept types cliniques de sévérité variable selon Silence [2]. Le type II (maladie de Porak et Durant) et le type III constituent des formes létales, non compatibles avec la vie. Cette maladie associe des signes squelettiques de sévérité variable et des signes extra squelettiques inconstants. Grâce à la prise en charge multidisciplinaire et à l'utilisation des biphosphonates, l’évolution des formes modérées de la maladie s'est beaucoup améliorée. Au Burkina Faso, très peu d’études ont été consacrées à la maladie. L'OI pose dans notre contexte d’énormes difficultés de prise en charge eu égard à l'insuffisance des moyens d'investigation, thérapeutiques et à l'indigence des parents. Nous rapportons dans notre étude quatre cas d'OI suivis aux Centres Hospitaliers Universitaires Charles de Gaulle (CHUP CDG) et Yalgado Ouédraogo (CHU YO) dont trois cas diagnostiqués dans la période néonatale et un cas à l’âge de 8 ans.

## Patient et observation

### Observation N°1

**Figure 1:**
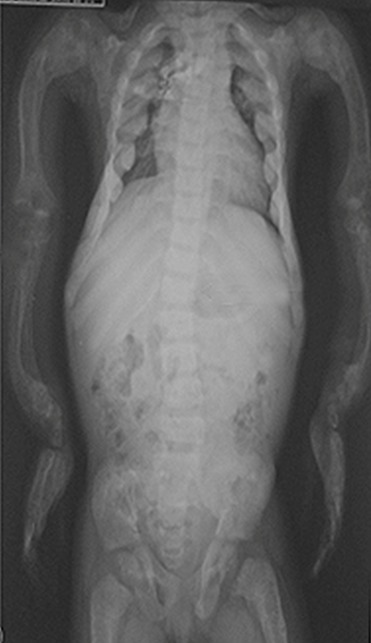
Radiographie thoraco-abdominale de face: transparence excessive des os, minceur de la corticale, déformation en arc des os des deux membres supérieurs, scoliose dorso-lombaire

Il s'agit d'une fillette âgée de 8 ans, reçue en consultation au CHUP CDG pour une déformation des membres inférieurs associée à un retard de croissance. Son développement moteur a été normal jusqu’à l’âge de 18 mois (âge de la marche). L'histoire clinique remonte à l’âge de deux ans marquée par l'installation progressive de déformations des membres, sans notion de traumatisme. L'examen à l'admission notait un bon état général, un trouble de l'audition et un retard staturo-pondéral avec un poids de 13,5 kg (soit < -3 Déviation Standard: DS) et une taille de 82,4 cm (< -3 DS). On notait également au niveau des yeux des sclérotiques bleutées; des déformations multiples du corps notamment une incurvation en « arc » des jambes et des bras, des pieds déformés en valgus, une déformation en tonneau du thorax et une déformation du dos. L'examen des autres appareils était normal. La radiographie du squelette a montré une déformation en arc des os au niveau des membres inférieurs, une scoliose dorsolombaire, une minceur des corticales et une transparence excessive des os avec des cals vicieux sur les os longs (Figure 1). L'audiométrie n'a pu être réalisée pour établir le type de trouble de l'audition. Sur le plan thérapeutique l'enfant n'a pas bénéficié d'un traitement médicamenteux par les biphosphonates. L’évolution est stationnaire et seule la station debout est actuellement possible avec soutien. Un fauteuil roulant a été prescrit pour ses déplacements.

### Observation N°2

**Figure 2:**
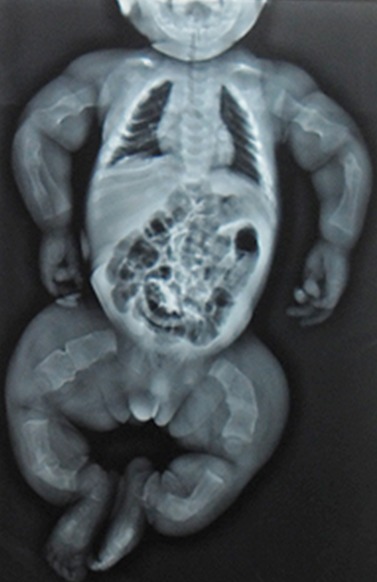
Radiographie thoraco-abdominale et des membres de face: déminéralisation de l'ensemble de la trame osseuse notamment des os longs. On note plusieurs fractures pathologiques avec des cals vicieux sur les os longs des membres

Il s'agit d'un nouveau-né reçu à 18 jours de vie en consultation au CHUP-CDG pour déformation des membres. Ce nouveau-né avait un retard de croissance avec un poids de 2 470 g (< - 3 DS), une taille de 41 cm (< - 3 DS); un périmètre crânien de 34 cm et un périmètre thoracique de 29 cm. L'examen clinique à l'admission a noté des sclérotiques bleutées, un syndrome poly malformatif caractérisé par une exophtalmie bilatérale, une dysmorphie faciale (visage triangulaire, un petit menton), une disjonction des sutures avec élargissement des fontanelles antérieure et postérieure, des cuisses larges, une incurvation en « arc » des membres inférieurs associée à des tuméfactions douloureuses et une hernie ombilicale. L'examen physique des autres appareils était normal. Sur le plan paraclinique, la radiographie thoraco-abdominale et des membres (Figure 2) montrait des déformations osseuses, une déminéralisation de l'ensemble de la trame osseuse avec des cals vicieux sur les os longs (fractures pathologiques). Le bilan pparaclinique à la recherche d'autres malformations avait objectivé une persistance du canal artériel à l’échographie doppler cardiaque. La prise en charge a consisté en un suivi régulier avec un soutien psychologique et des conseils aux parents. Il n'y a pas eu d'incident au cours de l’évolution jusqu'au 9e mois de vie où le nourrisson a été réadmis aux urgences médicales du CHUP CDG pour détresse respiratoire. Il est décédé au troisième jour d'hospitalisation dans un tableau d'insuffisance respiratoire.

### Observation N°3

**Figure 3:**
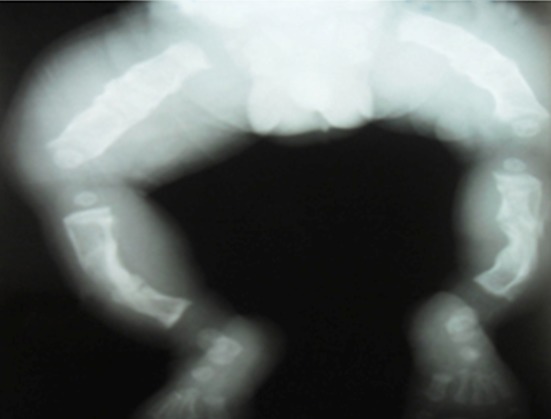
Radiographie de face des membres inférieurs: déformation en arc des os des jambes avec fracture du tiers inferieur du tibia gauche et du tiers supérieur du tibia droit. Déminéralisation osseuse diffuse

Ce nouveau-né de sexe masculin était admis au 9e jour de vie aux urgences médicales du CHUP-CDG pour fièvre et détresse respiratoire associées à des déformations des membres. L'examen clinique à l'admission a noté une mauvaise impression générale avec une détresse respiratoire et un syndrome infectieux. Le nouveau-né pesait 2900 g, avait un retard de croissance staturale avec une taille de 45 cm (< - 2 DS), un périmètre crânien de 35 cm et un périmètre thoracique de 31 cm. L'examen morphologique a montré un syndrome polymalformatif constitué d'une disjonction des sutures avec élargissement des fontanelles antérieure et postérieure; d'un visage triangulaire; des bras courts; des cuisses larges; des tuméfactions douloureuses des deux jambes qui étaient incurvées en « arc » et une volumineuse hernie ombilicale. L'examen physique des autres appareils était normal. La radiographie du squelette (Figure 3) a mis en évidence des déformations osseuses, une déminéralisation de l'ensemble de la trame osseuse avec des cals vicieux sur les os longs et une fracture des deux tibias. La prise en charge a nécessité une hospitalisation en soins intensifs. L’évolution a été marquée par la persistance du syndrome infectieux et de la détresse respiratoire. Le nouveau-né est décédé dans cet état au 6e jour d'hospitalisation.

### Observation N°4

**Figure 4:**
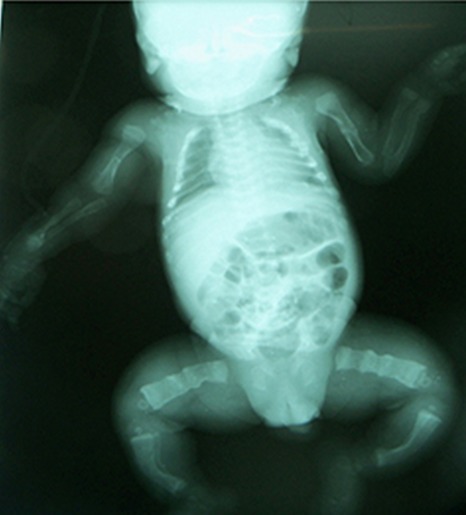
Radiographie thoraco-abdominale et des membres de face: déminéralisation de l'ensemble de la trame osseuse; fractures des os longs des membres avec cals vicieux; fracture du 1/3 supérieur du fémur gauche

Il s'agit d'un nouveau-né reçu au 2^e^ jour de vie aux urgences pédiatriques du CHU-YO pour une malformation congénitale des membres. Il est né avec un poids de 2500 g, une taille de 50 cm, un périmètre crânien de 32 cm et un périmètre thoracique de 32 cm. L'examen clinique à l'entrée avait noté une mauvaise impression générale, une détresse respiratoire et un syndrome infectieux. Le nouveau-né présentait également des sclérotiques bleutées; un syndrome polymalformatif avec une exophtalmie droite, une dysmorphie faciale (visage globalement triangulaire), une disjonction de la suture sagittale, des fontanelles béantes, des cuisses larges et les quatre membres déformés et incurvés en « arc ». Le bilan biologique a mis en évidence des stigmates d'une infection bactérienne avec une CRP (Protein Chain Reaction) à 50 mg/l. La radiographie thoraco-abdominale et des membres (Figure 4) a montré des déformations osseuses, une déminéralisation de l'ensemble de la trame osseuse avec des cals vicieux sur les os longs (fractures pathologiques), une fracture du tiers supérieur du fémur gauche. Le nouveau-né a bénéficié d'une prise en charge en soins intensifs. Une psychothérapie de soutien a été faite aux parents. L’évolution a été marquée par l'apparition d'un ictère et l'aggravation de la détresse respiratoire. Le nouveau-né est décédé en soins intensifs au 11^e^ jour d'hospitalisation.

## Discussion

### Aspects cliniques

L’état général était altéré chez deux nouveaux-nés (observations N° 3 et N° 4) qui présentaient également une détresse respiratoire. Cela corrobore les résultats d'observations faites par d'autres auteurs [1, 4, 5]. La détresse respiratoire est un signe généralement lié aux malformations thoraciques. L'installation précoce d'une détresse respiratoire témoigne de la sévérité du tableau clinique et présage d'une évolution le plus souvent fatale [1, 5] Les sclérotiques bleutées ont été retrouvées chez tous nos patients. C'est un signe retrouvé par plusieurs auteurs [1, 6–10]. Cette anomalie de la tunique externe de l’œil est caractéristique de l'ostéogenèse imparfaite. La dysmorphie faciale évidente chez deux de nos patients (observations N° 2 et N° 4) a été également décrite par Salimi au Maroc [8]. Elle est inconstante et favorisée par la disjonction des sutures et la malléabilité des os du crâne. Les déformations osseuses, plus marquées dans les types II et III de l'OI [11], sont fréquentes et font partie des signes cliniques évocateurs de la maladie. Elles étaient présentes à la naissance chez trois de nos patients (observations N° 2, N° 3 et N° 4) comme l'ont déjà décrit d'autres auteurs [1, 4, 5, 8]. Leur apparition a été progressive chez la première patiente (Observation N° 1) qui était classée comme un type I probable. La petite taille, décrite dans l'OI de type I et surtout de type III a été retrouvée chez le patient N° 3 parmi nos observations. Ce constat a été aussi fait par Salimi [8].

### Aspects para cliniques

L'examen clinique complété par la radiographie nous a permis de poser le diagnostic de l'OI en montrant les déformations osseuses, les multiples fractures (soit récentes, soit sous formes de cals osseux) et la déminéralisation de la trame osseuse. Cet examen radiologique a été la base du diagnostic dans d'autres études [1, 4, 8, 10]. Les fractures pathologiques, signes caractéristiques de l'ostéogenèse imparfaite sont fréquemment associées à des degrés variables à des déformations osseuses qui sont soit secondaires à des cals vicieux avec angulation, soit spontanées. Ces déformations ont été retrouvées chez tous nos patients. Chez les trois nouveau-nés (observations N° 2, N° 3 et N° 4), elles font évoquer des fractures intra utérines. Celles-ci ont été également retrouvées soit en période anténatale [1], soit en période post natale immédiate [5, 8]. Ces fractures touchent l'ensemble du squelette mais les os longs sont les plus concernés. Dans sa série de 68 observations, Greeley aux Etats-Unis [9] avait constaté 69 fractures de membres chez 22 patients et des fractures de côtes chez 15 patients. D'autres examens auraient permis une meilleure description de nos quatre observations. Il s'agit de l'ostéodensitométrie qui aurait montré un effondrement de la densité minérale osseuse; la biopsie osseuse avec examen anatomopathologique pour mettre en évidence une dysplasie du périoste et la biologie moléculaire à la recherche de l'anomalie génétique en cause. Ces examens n’étaient pas disponibles dans notre contexte de travail ou n'ont pas été réalisés.

### Aspects thérapeutiques

Le traitement a été essentiellement symptomatique chez tous nos patients. En effet, les biphosphonates qui ont révolutionné le traitement ne sont pas disponibles dans notre contexte de travail. Pourtant leur utilisation aurait été d'un bénéfice certain chez le seul enfant survivant (observation N° 1) de notre série. En effet, plusieurs études portant sur l'utilisation des biphosphonates ont montré des résultats satisfaisants [6, 10, 11]. Leur utilisation a permis l'obtention d'un gain considérable de la masse osseuse avec diminution drastique du nombre de fractures [12]. Le pamidronate est le biphosphonate le plus utilisé en pédiatrie. Selon le protocole proposé par Glorieux [13], le pamidronate est administré en perfusion lente de façon cyclique et plusieurs cycles de traitement sont nécessaires pour améliorer la densité minérale osseuse. La densité minérale et la réduction du taux de fractures, sont les deux critères majeurs d'efficacité thérapeutique mais il n'existe pas à l'heure actuelle de consensus quant aux critères d'arrêt thérapeutique. Selon Moulin, la normalisation de la densité minérale osseuse est un critère important et nécessite souvent trois à quatre années de traitement minimum [7].

### Aspects évolutifs

Dans notre étude, l’évolution a été fatale chez deux nouveau-nés après 15 jours (observation N° 3) et 13 jours de vie (observation N° 4). Il s'agit de formes probablement incompatibles avec la vie. Comme dans notre étude, Cissé [1] et Peyron [14] ont décrit une évolution défavorable à propos respectivement d'un cas et deux cas. Les deux autres patients de notre étude étaient suivis en ambulatoire jusqu'au décès au 9e mois de l'un d'eux (observation N° 3). L'absence de complications majeures chez la fillette de 8 ans (observation N° 1) pourrait présager d'une survie plus longue. Cependant, l’évolution à long terme peut être émaillée de complications à type d'aggravation du retard de croissance qui est lié à de probables fractures et à des tassements vertébraux [15] et ce d'autant plus que le traitement par les biphosphonates n'est toujours pas disponible. Des troubles de la statique vertébrale (lordose, cyphose, cyphoscoliose) liées à des anomalies de la croissance enchondrale peuvent également survenir [16]. Le pronostic dans l'observation N° 1 est donc réservé compte tenu de la sévérité des déformations osseuses, du risque de survenue d'autres fractures pathologiques et d'une insuffisance respiratoire inopinée.

## Conclusion

Notre étude montre d'une part la difficulté de faire un diagnostic étiologique de l'ostéogenèse imparfaite dans notre contexte de travail et d'autre part la nécessité d'améliorer la prise en charge par l'utilisation des biphosphonates dans cette maladie rare mais non exceptionnelle et handicapante. Sur le plan préventif, la pratique systématique de l’échographie fœtale en vue du diagnostic anténatal peut permettre une prise de décision précoce [17]. Le conseil génétique dans les situations à risque (antécédents personnels et/ou familiaux) est également nécessaire.
